# Tracheal Tumors: Clinical Practice Guidelines for Palliative Treatment and Follow-Up

**DOI:** 10.3389/or.2024.1451247

**Published:** 2024-09-18

**Authors:** Aleksandra Piórek, Adam Płużański, Magdalena Knetki-Wróblewska, Kinga Winiarczyk, Sylwia Tabor, Dariusz M. Kowalski, Maciej Krzakowski

**Affiliations:** Department of Lung Cancer and Thoracic Tumors, Maria Sklodowska-Curie National Research Institute of Oncology, Warsaw, Poland

**Keywords:** adenoid cystic carcinoma, follow-up, guideline, palliative treatment, squamous-cell carcinoma of the trachea, tracheal cancer

## Abstract

A substantial portion of patients with advanced cancer cannot be cured, regardless of the therapeutic methods employed. Hence, rational palliative causal treatment becomes crucial. Representative studies specifically addressing the exclusive palliative treatment of patients diagnosed with tracheal cancers have not been identified. In most studies, patients treated palliatively constituted a subset of the overall evaluated group. A thorough literature review was conducted, focusing on three types of palliative treatment: palliative radiotherapy, palliative surgical procedures, and systemic treatment for advanced disease. This review uniquely fills a significant gap in the existing literature by providing the first comprehensive and updated clinical practice guidelines specifically focused on the palliative treatment of tracheal tumors. The proposed guidelines emphasize the unique clinical challenges and treatment strategies pertinent to palliative care in tracheal tumors, which are not adequately covered in existing guidelines for other thoracic malignancies.

## Introduction

Primary tracheal tumors are rare, and their management is not definitely established, especially in palliative treatment. The most common types are squamous-cell carcinoma (SCC) and adenoid cystic carcinoma (ACC), which together account for more than two-thirds of primary tracheal tumors in adults [1]. Clinical symptoms may result from the obstruction of the airways (shortness of breath, wheezing, stridor), irritation and ulceration of the mucous membrane (cough, hemoptysis), or direct infiltration of adjacent structures (nerve paralysis, dysphagia). The disease is often diagnosed late due to the substantial functional reserves of the tracheal lumen [1, 2]. Presented symptoms are nonspecific and may lead to a misdiagnosis of asthma, chronic obstructive pulmonary disease, or bronchitis. The most common symptom of tracheal SCC is hemoptysis. The occurrence of hemoptysis usually leads to an earlier diagnosis of the tumor. However, hemoptysis occurs in less than 25% of patients in the early stages of the disease. The absence of symptoms often results in a delay in diagnosis, sometimes extending for several months [1]. The onset of hoarseness and shortness of breath typically indicates an advanced disease. Wheezing and stridor are the most common symptoms in the case of ACC. Diagnosis primarily relies on bronchoscopic examination, which allows for precise lesion localization, assessment of the extent of the disease, and obtaining a biopsy for histopathological examination. Several factors determine the prognosis of patients with primary tracheal tumors. Histological diagnosis of ACC [3–17], better performance status [10, 12, 18–20], and radical surgery [3, 4, 12, 13, 15, 21–25] have been identified as favorable prognostic factors. Most authors have advocated resection as the primary radical therapy of tracheal tumors, with or without adjuvant radiotherapy. Radiotherapy (RT) has also sometimes been advocated as the primary therapeutic modality [3]. What about advanced disease and palliative treatment? Distant metastases at the time of tracheal cancer diagnosis are rare. Gaissert et al. observed distant metastases in about 10% of patients with ACC and SCC of the trachea at diagnosis [6]. It should be noted that ACC has distinct growth dynamics and time to appearance of metastatic changes compared to SCC. In the initial years of observation, local treatment is characterized by high effectiveness (the rate of 5-year recurrence-free survival is 50%–75%); however, in subsequent years of observation, there is a steady increase in the number of patients with local recurrences or distant metastases. Approximately 10%–15% of patients remain disease free after 15 years of observation [26]. Distant metastases most commonly occur in the lungs [23, 27]. Patients with metastatic changes in the lungs have a better prognosis than those with metastases in other organs [26]. Lung metastases grow expansively and rarely cause clinical symptoms for many years [22]. Among a large group of patients (62 individuals) treated at the Mayo Clinic between 1972 and 2002, distant metastases were observed in 40.5% [23]. Among 15 patients with ACC, metastases mainly occurred in the lungs, brain, chest wall, and liver [23]. Initially, advanced disease is very rarely described. In another study, patients in the non-operative stage accounted for 23% (8 individuals), with only three initially diagnosed as stage IV [28]. In the subsequent two studies, patients in stage IV clinical advancement at diagnosis accounted for 8.3% (1 person) and 10% (3 people), respectively [22, 29]. In a 1996 study by Maziak et al., more than half of the evaluated patients were found to have exclusively metachronous distant metastases, with the most common location being the lungs. The authors showed that these changes often appear long after the initial diagnosis (12–300 months) and that patients with late spread have long survivals up to 7 years from diagnosis (mean survival period of 37 months). In various studies, authors also emphasized that metastatic lung changes in ACC cases can remain asymptomatic for a long time, highlighted the role of surgical treatment, and the need for long-term observation of patients [22, 23, 27].

The primary goal of therapeutic management is to control tumor growth, aiming to enhance both quality of life (QoL) and survival. Due to the limited number of dedicated clinical trials and the absence of reliable response predictors, the optimal strategy for palliative treatment remains uncertain.

In this review, we investigated treatment outcomes according to treatment modalities in patients with primary tracheal tumors, focusing attention on palliative care.

## Methodology

To develop the guidelines for the palliative treatment of tracheal tumors, we used an approach involving several stages. An expert panel consisting of seven specialists in clinical oncology, radiotherapy, and palliative care from the Department of Lung Cancer and Thoracic Tumors, Maria Sklodowska-Curie National Research Institute of Oncology in Warsaw, conducted a comprehensive literature review. We searched the PubMed and Google Scholar databases using relevant MeSH terms, including “tracheal neoplasms,” “Tracheal carcinoma,” “Tracheal tumors” to identify all available publications related to malignant tumors of the trachea. Where the entire manuscript or report was unavailable, abstracts were used to obtain the maximum possible information. The literature review included clinical trials, retrospective analyses, systematic reviews, meta-analyses, and case reports. Each piece of evidence was then evaluated for methodological quality and clinical relevance using the grading system developed by the Infectious Diseases Society of America-United States Public Health Service, classifying the evidence into levels and grades of recommendation. The recommendations were formulated based on the strength and consistency of the data and were refined through discussion and consensus-building among the panel members. This work constitutes a narrative review of the literature on the palliative treatment of tracheal tumors, based on available publications, and is not a structured systematic review.

## Management of Advanced/Metastatic Disease

Control of tumor growth is the goal of therapeutic management, aiming to improve both QoL and survival. The best strategy for palliative treatment is unknown because of the low number of dedicated clinical trials and lack of response predictors. Such information should be provided to the patient. Watchful waiting may be considered in asymptomatic and slowly progressing ACC patients.

## Systemic Anti-Tumor Therapy

Systemic anticancer therapies in patients with advanced tracheal tumors mainly include chemotherapy (CTH), but there are also cases where targeted therapies or immunotherapy have been used. These systemic therapies should be discussed within expert multidisciplinary teams and reserved for patients with morphologically progressive tumors or symptoms. So far, no specific phase III clinical trials exclusively for tracheal tumors have been published, so most evidence comes mainly from retrospective analyses and sporadically from randomized phase II trials or subgroup analyses of phase III trials, where the majority of patients were diagnosed with head and neck ACC.

### Chemotherapy

#### ACC

The biology of ACC is indolent, and indications for chemotherapy are not clearly defined; however, it appears that chemotherapy can reduce symptoms associated with significant disease progression [30, 31]. Prolongation of overall survival has not been clearly demonstrated, and patients with recurrent disease have been shown to have lower efficacy with respect to surgical re-treatment or radiation therapy [32]. For years, cyclophosphamide, doxorubicin, cisplatin, 5-fluorouracil, etoposide, and mitoxantrone have been considered active cytostatic agents for this diagnosis [30, 33, 34]. In patients with symptoms of advanced disease, multi-agent chemotherapy may be considered. The regimen most commonly used is the CAP regimen (cisplatin, doxorubicin, cyclophosphamide) regimen, with objective response rates of approximately 25% and response times of 6–77 months [30]. In cases of low disease dynamics, cytostatic monotherapy can be implemented - with an expected response time of 5–20 months and an objective response rate of approximately 15% [35]. Monotherapy is reasonable given the slow dynamics of ACC and a more favorable safety profile [30, 31, 35]. Rizk et al. presented an analysis of 205 patients with advanced and recurrent salivary gland tumors (clinical data were collected based on published information and obtained from the authors), with various histology (non-squamous carcinoma predominated) [36]. In multivariate analysis, platinum-based chemotherapy was found to be an independent favorable prognostic factor for OS - HR 0.56 (95% CI 0.36–0.89; p < 0.01) [36]. On the other hand, it appears that primary tracheal ACC may have a much less dynamic course than primary bronchial ACC, making the indication for chemotherapy even more questionable [37].

#### SCC

Squamous cell carcinoma is the predominant histology of primary tracheal carcinomas, typical of smokers and men over 60 years of age. Due to comorbidities and clinical characteristics, surgical treatment or even radical chemoradiotherapy is not always feasible. The best supportive care or palliative chemotherapy remains an option, but this approach significantly worsens the prognosis [38]. Manninen et al. published the results of a group of 12 patients treated with cyclophosphamide alone or in combination with vincristine - no objective response to treatment was observed, and survival was shorter than in patients receiving palliative radiation therapy, including brachytherapy [14]. Case reports indicate the feasibility of regimens based on cytostatic agents such as paclitaxel, carboplatin, cisplatin, and 5-fluorouracil [39–41]. Treatment combining platinum-based chemotherapy with immune checkpoint inhibitors - toripalimab, nivolumab - remains an area of discussion and provided clinical trials NCT04716751, NCT05964101, NCT02834013 [42, 43].

### Recommendations

• Consider multi-agent chemotherapy for patients with advanced ACC exhibiting significant disease progression and symptoms [II, B].
• Use cytostatic monotherapy for patients with ACC who have low disease dynamics [II, B].
• Consider platinum-based chemotherapy as a palliative option for patients with advanced SCC who are not candidates for radical treatment [II, B].
• Consider enrolling in clinical trials investigating the combination of chemotherapy with immune checkpoint inhibitors for patients with advanced SCC [II, B].

### Targeted Therapies

#### ACC

As first inhibitors of tyrosine kinase against the c-KIT receptor were investigated. In two phase 2 studies, 26 patients were treated with imatinib. Objective responses were not achieved; in most patients, disease progression occurred within 6 months [44]. With sunitinib, a kinase inhibitor targeting several signaling pathways, objective responses were also not achieved, and the percentage of reported toxicities such as mucositis, fatigue, and hypophosphatemia affected 50% of patients [45]. Based on the studies that were conducted, it can be inferred that blocking the c-KIT pathway is not effective in patients with ACC. Another pathway evaluated as potentially effective in treating ACC patients is the EGFR pathway. Unfortunately, the efficacy of therapy with gefitinib, cetuximab, or lapatinib has also not been confirmed [21, 46, 47].

In a phase 2 study involving 28 patients with recurrent or metastatic ACC, the multi-kinase inhibitor lenvatinib was used, resulting in partial response (PR) in 3 patients and disease stabilization in 20 patients. The median overall survival (OS) and progression-free survival (PFS) were 27 months and 9.7 months, respectively. However, treatment was associated with significant toxicity, and 24 patients required dose reduction [48]. Similar results were obtained by Tchekmedyian et al. - 15.6% of 32 patients achieved partial response. Treatment had to be discontinued in 18 patients because of toxicity, mainly arterial hypertension and painful oral mucosal dryness [49].

Another potential molecular target in ACC treatment is the PI3K/PTEN/mTOR pathway. Everolimus was evaluated in a phase 2 study involving 34 patients with unresectable ACC. None of the patients achieved complete response (CR) or PR, while 27 patients (79.4%) achieved stable disease (SD) [50].

Mutations in NOTCH1 are another evaluated disorder associated with more aggressive disease, increased risk of distant metastasis, and shorter survival time in ACC patients [51]. In a Phase 1 study using the pan-notch inhibitor, LY3039478 was conducted on a group of 22 patients; one patient achieved PR, and four patients achieved SD. Reported adverse events included diarrhea, vomiting, skin dryness, and dryness in the oral mucosa [52].

Table 1 presents some of the targeted drugs evaluated in phase 2 studies for advanced ACC [53].

**TABLE 1:** Phase 2 trials of targeted agents in ACC [53].

Agent	Molecular target	N	PR	SD
Imatinib [54]	c-KIT CD117	10	0	2 (20%)
Lapatinib [21]	HER-2, EGFR	19	0	15 (79%)
Cetuximab [47]	EGFR	23	0	20 (87%)
Bortezomib [55]	Proteosome inhibitor	25	0	16 (64%)
Sunitinib [45]	VEGFR, c-KIT, PDGFR	13	0	11 (62%)
Ewerolimus [50]	PI3K/PTEN/mTOR	34	0	27 (79.4%)
Gefitinib [46]	EGFR	19	0	13 (68%)
Axitinib [56]	VEGFR, KIT, PDGFR	33	3 (9%)	25 (76%)
Dasatinib [57]	c-KIT, PDGFR	40 (ACC)	1 (2.5%)	20 (50%)
Nintedanib [58]	VEGFR, FGFR, PDGFR	20	0	15 (75%)
Vorinostat [59]	Histone deacetylase inhibitor	30	2 (6.7%)	27 (90%)
Dovitinib [60]	VEGFR, PDGFR, FGFR, c-KIT, CSF-1R	34	2 (6%)	22 (65%)
Lenwatinib [48]	FGFR1, VEGFR2, c-KIT, RET, PDFR-α PDGFR-β	28	3 (11.5%)	20 (71%)

CSF-1R, colony stimulating factor-1 receptor; EGFR, epidermal growth factor receptor; FGFR, fibroblast growth factor receptor; HER-2, human epidermal receptor-2; PDGFR, platelet-derived growth factor receptor; PI3K/PTEN/mTOR, phosphoinositide 3-kinase (PI3K)/phosphatase and tensin homologue deleted on chromosome ten (PTEN)/mammalian target of rapamycin (mTOR) pathway; PR, partial response; RET, rearranged during transfection; SD, stable disease; VEGFR, vascular endothelial growth factor receptor.

#### SCC

Squamous cell carcinoma of the trachea, similar to other SCCs, presents limited molecular characteristics that can be exploited in targeted therapy. Overexpression and mutations in EGFR and EGFR inhibitors, such as gefitinib and cetuximab, have been explored in clinical settings, though their efficacy in tracheal SCC specifically requires further validation [61]. Alterations in FGFR, including mutations and overexpression, can also lead to the activation of signaling pathways that promote tumor cell growth and survival. FGFR inhibitors are being investigated as potential therapies in SCC, showing promising results in inhibiting tumor growth [61]. The PI3K/AKT/mTOR pathway is critical for regulating cell growth, proliferation, and survival, and its activation is often observed in various cancers, including SCC. PI3K inhibitors, which block this pathway, can reduce tumor cell growth and survival, making them promising candidates for SCC therapy. Unfortunately, early phase studies evaluating treatment with PI3K inhibitors found no significant clinical benefit from the treatment, with a response rate that does not exceed 5% and medial progressive-free survival similar to what was obtained from docetaxel [61].

Personalised medicine approaches in tracheal tumors are ultimately hampered by the limited amount of available research to identify reliable, validated molecular markers with prognostic or predictive value. However, the recent identification of molecular alterations may lead to consideration of next-generation sequencing (NGS) and—in an off-label setting—the use of targeted agents for the treatment of refractory tracheal tumors, especially ACC. Thus, NGS may be recommended for later lines of therapy, when standard treatment options are exhausted, and there is a need to explore targeted therapies based on the specific genetic profile of the tumor.

### Recommendations

• Consider targeted therapies based on molecular profiling for patients with advanced tracheal tumors [III, B].
• Investigate clinical trials for new and emerging targeted therapies in tracheal tumor treatment [II, B].

### Immunotherapy

#### ACC

ACC is a tumor characterized by low expression of programmed death–ligand 1 (PD-L1) receptors on the surface of tumor cells combined with poor immune infiltration and antigen presentation [62, 63]. In a retrospective analysis of histopathological material from patients with tracheal tumors, no expression of PD-L1 receptors was found in any of the 14 analyzed cases of ACC [64]. As for tumor mutational burden (TMB), only 1% of patients diagnosed with ACC meet the criteria for high TMB, which is more than ten mutations per megabase [65].

Pembrolizumab demonstrated its activity in patients diagnosed with advanced PD-L1-positive salivary gland carcinoma, as shown in the phase Ib clinical trial KEYNOTE-028. The study included only two patients diagnosed with ACC, who showed a better ORR than results from studies using sorafenib or dovitinib; however, this was not demonstrated for survival parameters such as OS or PFS [66]. On the contrary, in a phase II study comparing the use of pembrolizumab with or without hypofractionated locoregional radiotherapy in metastatic or recurrent ACC, no statistically significant differences were observed between the arms in terms of ORR, PFS, or OS. An objective response outside of the irradiated region was observed in none of the patients [67].

According to the National Comprehensive Cancer Network (NCCN) recommendations for head and neck cancers, pembrolizumab as the only checkpoint inhibitor agent is recommended in the systemic therapy of patients diagnosed with salivary gland tumors who have microsatellite instability (MSI-H), mismatch repair deficiency (dMMR) or high TMB [68]. This recommendation is based on data from the KEYNOTE-158 clinical trial, in which three patients with salivary gland cancer and TMB high status participated without detailed histological subtype differentiation [69].

Currently, we do not have data from phase I, II or III clinical trials using immunotherapy in tracheal ACC. The only available case report concerns a patient in whom the use of durvalumab in adjuvant treatment after chemoradiotherapy resulted in 15 – months recurrence free period [70].

#### SCC

The biological characteristics and histopathological features of tracheal SCC appear to be similar to one of the most common human malignancies which is lung cancer [61]. In the previously cited study, PD-L1 expression was observed in 3 out of 4 analyzed tissue samples from patients with SCC [64], which clearly distinguishes this histological subtype from ACC.

The above statements are confirmed by case reports available in the literature. In one of them, the use of pembrolizumab in a patient who had exhausted available therapeutic options resulted in a sustained complete response to the treatment for a period of 11 months. The estimated PD-L1 expression in this SCC case was greater than 90% [71]. On the other hand, the use of nivolumab therapy in a patient with locally advanced tracheal SCC, after numerous surgical interventions and systemic therapies, allowed for achieving local disease control and significant clinical improvement. The bronchoscopy performed after 7 months of treatment showed remission of a tracheal tumor. In this case, the presence of PD-L1 receptors on the cancer cells was also demonstrated [72].

### Recommendations

• There is insufficient scientific evidence to justify the use of checkpoint inhibitors in advanced primary tracheal ACC [V, C].
• Taking into consideration clinical and histological premises, as well as data from case reports, it appears that the use of immunotherapy may be a beneficial therapeutic option for patients with advanced tracheal SCC who exhibit PD-L1 expression [V, B].

## Locoregional Palliative Therapy

### Surgery

For some patients who present with metastatic disease, palliative resection can be used to relieve airway obstruction in cases where a tracheostomy is not feasible [4, 73]. The efficacy and safety of palliative surgical procedures for patients with obstruction due to ACC was presented in the work of Lee et al. [22]. Endobronchial treatment is palliative, but it may be assumed that it also can prolong life [74]. It should, in addition to surgery, medication, and radiotherapy, be available in any combination for these patients [74], but the prognosis for patients who cannot undergo radical treatment is generally poor.

Restoration of the lumen can be achieved through various endoscopic techniques, including dilation (mechanical endoscopic dilation of stenosis), laser vaporization, electrocoagulation, cryotherapy, photodynamic therapy, or argon coagulation [1, 75]. In most cases, the mentioned methods provide improvement; however, they often require repeated interventions and do not ensure a lasting effect. In non-operable cases, improvement in airway patency can be achieved using a silicone or self-expanding stent. A satisfactory palliative effect can be achieved in 80%–90% of appropriately qualified patients [1]. Literature reports on self-expanding intratracheal stents, however, indicate that despite efforts to improve the material used to create a functional scaffold, a limitation of the method is granulation within the tracheal lumen induced by the introduced foreign body, which may lead to an increase in the length of the stenosis. Additionally, among other disadvantages, stent displacement and the formation of esophageal and vascular fistulas are cited [2]. Self-expanding metal stents can be applied in patients with an expected survival of 3–6 months [2]. They are not suitable for non-operable patients diagnosed with ACC. Prolonged survivals are observed in these patients despite advanced disease [75].

### Recommendations

• Consider palliative surgical treatment for patients with pronounced symptoms of airway obstruction when radical treatment is not feasible [III, B].
• Utilize various endoscopic techniques, such as dilation, laser vaporization, electrocoagulation, cryotherapy, photodynamic therapy, or argon coagulation, to restore the airway lumen [III, B].
• Reserve self-expanding metal stents for patients with an expected survival of 3–6 months, particularly those diagnosed with ACC [IV, B].

### Radiotherapy

Palliative radiotherapy for tracheal cancer is essential for managing symptoms caused by localized tumor growth in patients who are not eligible for radical treatment. The primary aim of palliative radiotherapy is to control severe symptoms such as dyspnea, hemoptysis, and pain by reducing tumor size and its obstructive effects on the airway. A favorable palliative response can be achieved in 75% of treated patients. Studies involving palliative RT have observed a good palliative effect in terms of alleviating obstruction and reducing the severity of symptoms [11, 12]. The percentage of patients showing improvement after 3 months of RT was 56.3% for shortness of breath and 72.2% for hemoptysis, with a mean duration of response reaching 12.5 months [11]. The median survival for patients undergoing palliative radiotherapy is approximately 6 months, with a 1-year overall survival rate of 31.6% [74]. Treatment plans vary, but the median dose typically is around 30 Gy, delivered in a range of fractions. Although the prognosis remains poor, this treatment modality is crucial in offering palliative care to optimize patient outcomes and manage the severe symptoms associated with advanced tracheal cancer.

### Recommendations

• Consider palliative radiotherapy for patients with localized tumor growth who are not candidates for radical treatment [III, B].
• Common indications for palliative radiotherapy include hemoptysis, pain, shortness of breath, and cough [III, B].

### Radiochemotherapy

The combined radiochemotherapy (RCTH) approach is an established method for the radical treatment of many locally advanced cancers. The biological basis for combining both methods lies in increasing the effectiveness of local and regional cures while reducing the risk of distant metastases. RCTH is also used as part of organ-sparing procedures, serving as an alternative to extensive surgical interventions. Concurrent RCTH with cisplatin is the treatment of choice for patients with locally advanced head and neck cancers. Both concurrent and sequential RCTH have been shown to be superior to standalone RT in the treatment of locally advanced lung cancers, establishing it as the standard of care in these cases. Data regarding the combination of CTH and RT for tracheal tumors are very limited, with only individual case reports and retrospective studies involving small groups of patients. RCTH is primarily used in situations where the patient cannot undergo radical surgical treatment. There is no data on the use of RCTH in the palliative treatment of tracheal tumors. Below, we present the available studies on RCTH in the treatment of tracheal tumors.

Joshi et al. (2010) described a patient with unresectable SCC who achieved long-term disease-free survival after RCTH [76]. Similar results were obtained by Videtic et al. (2003), where the combination of cisplatin and etoposide with RT led to a complete tumor response [77]. Allen et al. (2007) demonstrated the efficacy of RCTH in patients with unresectable ACC of the trachea, where treatment with carboplatin and paclitaxel, along with 66 Gy of RT, resulted in a complete response with no residual tumor [78]. Yathiraj et al. (2017) reported that patients with locally advanced, unresectable upper tracheal SCC treated with cisplatin and 3D-RCTH remained disease-free for 36 months [79]. Larnaudie et al. (2022) reviewed that systemic treatments were realized in RCTH as neoadjuvant or concomitant with radiotherapy [80]. In the majority of studies, platinum salt derivatives, primarily cisplatin, were used. Cisplatin was administered either at a dose of 100 mg/m^2^ every 3 weeks or 40 mg/m^2^ weekly and/or in combination with other molecules. In other combinations, a case report used carboplatin with paclitaxel [80]. Toxicity most commonly involves acute esophageal reactions.

### Recommendation

• There are no available data on the use of RCTH in the palliative treatment of tracheal tumors. Therefore, caution is advised when considering RCTH as a therapeutic option in this patient group, and treatment decisions should be made on an individual basis, guided by the patient’s clinical status and the available evidence [V, C].

### Novel and Investigational Therapies in Tracheal Carcinoma

Recent advancements in radiation therapy have introduced several novel modalities that may offer potential benefits in highly selective cases of tracheal carcinoma, though they remain largely investigational. One such approach is Stereotactic Body Radiation Therapy (SBRT), which delivers highly precise doses of radiation over fewer treatment sessions, targeting tumors with minimal exposure to surrounding healthy tissue. SBRT is particularly appealing in scenarios where surgical resection is not feasible, and studies have demonstrated its effectiveness in controlling local lung tumors. However, there are significant concerns regarding toxicity, especially when treating ultra-central tumors—those located very close to critical structures like the trachea, main bronchi, or esophagus [81].

Proton therapy is characterized by its high precision in delivering radiation doses, minimizing damage to surrounding healthy tissues, which is particularly important for tumors near critical structures like the trachea [82]. Preliminary studies suggest that proton therapy could be beneficial in cases of tracheal ACC, particularly when conventional photon therapy might lead to excessive toxicity [83–85].

Similarly, neutron therapy, which has a higher linear energy transfer (LET) compared to protons, has been explored, especially for radioresistant tumors like ACC. However, the severe late toxicities associated with neutron therapy have limited its use, and it is currently available in only a few centers worldwide [83, 85].

Another emerging approach is carbon ion radiotherapy (CIRT), which combines the benefits of proton therapy with greater radiobiological effectiveness. CIRT offers promising outcomes in treating difficult cases, including tracheal cancer, though it is still available only at a limited number of centers and requires further clinical research [10, 85].

The use of these advanced radiotherapy methods is particularly intriguing in cases where traditional treatment options are inadequate or impossible. Further studies and clinical trials are needed to fully establish their efficacy and safety in tracheal carcinoma treatment.

### Recommendation

• The use of advanced radiotherapy techniques, such as SBRT, proton therapy, neutron therapy, and CIRT, may be considered in the treatment of tracheal carcinoma in highly selective cases, particularly when traditional treatment options are inadequate or infeasible. Due to the limited clinical trial data and potential risks of toxicity, their application should be restricted to specialized centers with the necessary expertise and conducted within controlled clinical trials [IV, C].

### Long-Term Survivorship

Recurrence in patients treated for tracheal cancer is common and occurs in almost 25%–50% of patients after local treatment. The schedule of follow-up after complete palliative treatment depends on the possibilities of further therapies that may be limited, especially in patients receiving prior adjuvant or palliative radiation therapy.

Taking into account the smoking-dependent nature of SCC, a pattern of observation may be similar to that of a group of patients diagnosed with squamous cell lung cancer. During the surveillance period, another smoking-related cancer may be diagnosed. The surveillance examination may include serial CT scans and endoscopic procedures over the next 5 years.

For those with ACC there is a steady increase in the risk of local recurrences or distant metastases in subsequent years of observation. Therefore, in patients with ACC, the follow-up period should exceed 5 years.

### Recommendations

• The schedule of follow-up depends on the possibilities of further therapies.
• The surveillance examination may include serial CT scans and endoscopic procedures.
• The follow-up schedule is not defined.
• In patients amenable for further therapies consider serial CT scans and endoscopic procedures every 6–12 months up to minimum 5 years of observation.
• Due to the increasing risk of late relapse in patients with ACC a, surveillance over 5 years is recommended.

## Limitations

Our manuscript has several limitations that should be considered. First, although we conducted a comprehensive literature review, we primarily relied on publications in English available in the PubMed and Google Scholar databases, which may lead to the omission of significant studies published in other languages or less accessible sources. Second, our recommendations are based on available evidence, which often includes studies of varying methodological quality, such as retrospective analyses and case reports that do not provide as strong evidence as randomized clinical trials. The rarity of tracheal tumors limits the availability of high-quality evidence, forcing us to rely heavily on lower-level evidence. Third, the grades of recommendation were determined by authors from a single oncology center in Poland. While these experts possess significant knowledge and experience, the panel consisted solely of experts from one country, and there may be inherent biases related to local clinical practices and healthcare infrastructure that could influence the guidelines. The process used to determine the grades of recommendation involved expert discussion, an informal method of achieving consensus. The lack of a formal method may affect the perception of the recommendations.

## Summary

The prognosis for patients who cannot undergo radical treatment is poor. Available literature data and our own observations do not allow for definitive conclusions regarding the use of systemic treatment in the group of patients diagnosed with primary tracheal tumors. It is crucial to remember the adverse effects of treatment, making appropriate qualification for systemic treatment essential. Available tools, such as assessing the general condition using the Karnofsky Performance Status Scale or the World Health Organization (WHO) scale, as well as evaluating clinical symptoms and laboratory tests, form the basis for this qualification. These assessments help in identifying patients who might benefit from systemic treatments despite the poor prognosis. Besides systemic treatments, palliative radiotherapy and surgery are integral components of managing advanced tracheal cancer. Radiotherapy is effective in alleviating symptoms such as shortness of breath, hemoptysis, and cough in approximately 75% of patients. It helps control localized tumor growth and improves the patient’s quality of life. Palliative surgical procedures, including techniques like dilation, laser vaporization, electrocoagulation, cryotherapy, photodynamic therapy, and the use of self-expanding stents, can restore airway patency and relieve obstruction. These interventions, although often requiring repetition, provide significant symptomatic relief. Therefore, even though the overall outlook remains challenging, optimizing systemic treatment protocols and integrating radiotherapy and surgical interventions based on thorough patient assessment can improve palliative care outcomes, offering a multifaceted approach to managing severe symptoms associated with advanced tracheal cancer.

Future research directions should focus on conducting dedicated clinical trials for the palliative treatment of tracheal tumors to better understand the efficacy of various therapeutic methods. It is also important to develop new technologies and techniques that can improve the outcomes of palliative care. The potential clinical implications of our recommendations include better symptom control, improved quality of life for patients, and the optimization of therapeutic approaches tailored to the specific needs of patients with tracheal tumors. Implementing our guidelines may also contribute to the standardization of palliative care and increase awareness of the unique challenges associated with treating these rare tumors.

## References

[1] Macchiarini P. Primary Tracheal Tumours. The Lancet Oncol (2006) 7:83–91. 10.1016/s1470-2045(05)70541-6 16389188

[2] Madariaga MLL Gaissert HA. Overview of Malignant Tracheal Tumors. Ann Cardiothorac Surg (2018) 7:244–54. 10.21037/acs.2018.03.04 29707502 PMC5900094

[3] Webb BD Walsh GL Roberts DB Sturgis EM. Primary Tracheal Malignant Neoplasms: The University of Texas MD Anderson Cancer Center Experience. J Am Coll Surgeons (2006) 202:237–46. 10.1016/j.jamcollsurg.2005.09.016 16427548

[4] Gaissert HA Grillo HC Shadmehr MB Wright CD Gokhale M Wain JC Long-Term Survival after Resection of Primary Adenoid Cystic and Squamous Cell Carcinoma of the Trachea and Carina. The Ann Thorac Surg (2004) 78:1889–97. 10.1016/j.athoracsur.2004.05.064 15560996

[5] Regnard JF Fourquier P Levasseur P. Results and Prognostic Factors in Resections of Primary Tracheal Tumors: A Multicenter Retrospective Study. The French Society of Cardiovascular Surgery. J Thorac Cardiovasc Surg (1996) 111:808–13. 10.1016/s0022-5223(96)70341-0 8614141

[6] Wen J Liu D Xu X Chen D Chen Y Sun L Nomograms for Predicting Survival Outcomes in Patients With Primary Tracheal Tumors: A Large Population-Based Analysis. Cancer Management Res (2018) 10:6843–56. 10.2147/cmar.s186546 PMC6294060 30588090

[7] Bhattacharyya N. Contemporary Staging and Prognosis for Primary Tracheal Malignancies: A Population-Based Analysis. Otolaryngol Head Neck Surg (2004) 131:639–42. 10.1016/j.otohns.2004.05.018 15523440

[8] Zhengjaiang L Pingzhang T Dechao Z Reddy-Kolanu G Ilankovan V. Primary Tracheal Tumours: 21 Years of Experience at Peking Union Medical College, Beijing, China. J Laryngol Otol (2008) 122:1235–40. 10.1017/s0022215108001710 18331654

[9] Urdaneta AI Yu JB Wilson LD. Population Based Cancer Registry Analysis of Primary Tracheal Carcinoma. Am J Clin Oncol (2011) 34:32–7. 10.1097/coc.0b013e3181cae8ab 20087156

[10] Napieralska A Miszczyk L Blamek S. Tracheal Cancer - Treatment Results, Prognostic Factors and Incidence of Other Neoplasms. Radiol Oncol (2016) 50:409–17. 10.1515/raon-2016-0046 27904449 PMC5120581

[11] Makarewicz R Mross M. Radiation Therapy Alone in the Treatment of Tumours of the Trachea. Lung Cancer (1998) 20:169–74. 10.1016/s0169-5002(98)00018-x 9733051

[12] Hetnal M Kielaszek-Cmiel A Wolanin M Korzeniowski S Brandys P Małecki K Tracheal Cancer: Role of Radiation Therapy. Rep Pract Oncol & Radiother (2010) 15:113–8. 10.1016/j.rpor.2010.08.005 PMC3863152 24376936

[13] Honings J van Dijck JA Verhagen AF van der Heijden HF Marres HA. Incidence and Treatment of Tracheal Cancer: A Nationwide Study in the Netherlands. Ann Surg Oncol (2007) 14:968–76. 10.1245/s10434-006-9229-z 17139460

[14] Manninen MP Pukander JS Flander MK Laippala PJ Huhtala HS Karma PH. Treatment of Primary Tracheal Carcinoma in Finland in 1967-1985. Acta Oncologica (1993) 32:277–82. 10.3109/02841869309093595 8323765

[15] Licht PB Friis S Pettersson G. Tracheal Cancer in Denmark: A Nationwide Study. Eur J Cardio-Thoracic Surg (2001) 19:339–45. 10.1016/s1010-7940(01)00597-8 11251276

[16] Yang KY Chen YM Huang MH Perng RP. Revisit of Primary Malignant Neoplasms of the Trachea: Clinical Characteristics and Survival Analysis. Jpn J Clin Oncol (1997) 27:305–9. 10.1093/jjco/27.5.305 9390206

[17] Piorek A Pluzanski A Teterycz P Tabor S Winiarczyk K Knetki-Wroblewska M Clinicopathological Characteristics of Patients with Primary Tracheal Tumors: Analysis of Eighty-Nine Cases. Thorac Cancer (2024). 15:878–83. 10.1111/1759-7714.15231 38429910 PMC11016403

[18] Chao MW Smith JG Laidlaw C Joon DL Ball D. Results of Treating Primary Tumors of the Trachea With Radiotherapy. Int J Radiat Oncology*Biology*Physics (1998) 41:779–85. 10.1016/s0360-3016(98)00120-5 9652838

[19] Jeremic B Shibamoto Y Acimovic L Milisavljevic S. Radiotherapy for Primary Squamous Cell Carcinoma of the Trachea. Radiother Oncol (1996) 41:135–8. 10.1016/s0167-8140(96)01797-5 9004356

[20] Mornex F Coquard R Danhier S Maingon P El Husseini G Van Houtte P. Role of Radiation Therapy in the Treatment of Primary Tracheal Carcinoma. Int J Radiat Oncology*Biology*Physics (1998) 41:299–305. 10.1016/s0360-3016(98)00073-x 9607345

[21] Agulnik M Cohen EW Cohen RB Chen EX Vokes EE Hotte SJ Phase II Study of Lapatinib in Recurrent or Metastatic Epidermal Growth Factor Receptor And/or erbB2 Expressing Adenoid Cystic Carcinoma and Non Adenoid Cystic Carcinoma Malignant Tumors of the Salivary Glands. J Clin Oncol (2007) 25:3978–84. 10.1200/jco.2007.11.8612 17761983

[22] Lee JH Jung EJ Jeon K Koh WJ Suh GY Chung MP Treatment Outcomes of Patients With Adenoid Cystic Carcinoma of the Airway. Lung Cancer (2011) 72:244–9. 10.1016/j.lungcan.2010.08.011 20828861

[23] Molina JR Aubry MC Lewis JE Wampfler JA Williams BA Midthun DE Primary Salivary Gland-type Lung Cancer: Spectrum of Clinical Presentation, Histopathologic and Prognostic Factors. Cancer (2007) 110:2253–9. 10.1002/cncr.23048 17918258

[24] Mallick S Benson R Giridhar P Rajan Singh A Rath GK. Demography, Patterns of Care and Survival Outcomes in Patients With Malignant Tumors of Trachea: A Systematic Review and Individual Patient Data Analysis of 733 Patients. Lung Cancer (2019) 132:87–93. 10.1016/j.lungcan.2019.04.017 31097099

[25] Piorek A Pluzanski A Knetki-Wroblewska M Winiarczyk K Tabor S Teterycz P Treatment Outcomes of Patients with Primary Tracheal Tumors - Analysis of a Large Retrospective Series. BMC Cancer (2024) 24:686. 10.1186/s12885-024-12450-z 38840114 PMC11155021

[26] Papaspyrou G Hoch S Rinaldo A Rodrigo JP Takes RP van Herpen C Chemotherapy and Targeted Therapy in Adenoid Cystic Carcinoma of the Head and Neck: A Review. Head Neck (2011) 33:905–11. 10.1002/hed.21458 20652885

[27] Yang H Yao F Tantai J Zhao Y Tan Q Zhao H. Resected Tracheal Adenoid Cystic Carcinoma: Improvements in Outcome at a Single Institution. The Ann Thorac Surg (2016) 101:294–300. 10.1016/j.athoracsur.2015.06.073 26431923

[28] Hu MM Hu Y He JB Li BL. Primary Adenoid Cystic Carcinoma of the Lung: Clinicopathological Features, Treatment and Results. Oncol Lett (2015) 9:1475–81. 10.3892/ol.2015.2859 25663934 PMC4314995

[29] Zhu F Liu Z Hou Y He D Ge X Bai C Primary Salivary Gland-Type Lung Cancer: Clinicopathological Analysis of 88 Cases from China. J Thorac Oncol (2013) 8:1578–84. 10.1097/jto.0b013e3182a7d272 24389442

[30] Laurie SA Ho AL Fury MG Sherman E Pfister DG. Systemic Therapy in the Management of Metastatic or Locally Recurrent Adenoid Cystic Carcinoma of the Salivary Glands: A Systematic Review. The Lancet Oncol (2011) 12:815–24. 10.1016/s1470-2045(10)70245-x 21147032

[31] Zupancic M Nasman A Friesland S Dalianis T. Adenoid Cystic Carcinoma, Clinical Presentation, Current Treatment and Approaches towards Novel Therapies. Anticancer Res (2024) 44:1325–34. 10.21873/anticanres.16929 38537991

[32] Wang Y Cai S Gao S Xue Q Mu J Gao Y Tracheobronchial Adenoid Cystic Carcinoma: 50-Year Experience at the National Cancer Center, China. The Ann Thorac Surg (2019) 108:873–82. 10.1016/j.athoracsur.2019.03.065 31026435

[33] Creagan ET Woods JE Rubin J Schaid DJ. Cisplatin-based Chemotherapy for Neoplasms Arising From Salivary Glands and Contiguous Structures in the Head and Neck. Cancer (1988) 62:2313–9. 10.1002/1097-0142(19881201)62:11<2313::aid-cncr2820621110>3.0.co;2-4 3179947

[34] Lagha A Chraiet N Ayadi M Krimi S Allani B Rifi H Systemic Therapy in the Management of Metastatic or Advanced Salivary Gland Cancers. Head & Neck Oncol (2012) 4:19–57. 10.1186/1758-3284-4-19 PMC3414773 22558945

[35] Andry G Hamoir M Locati LD Licitra L Langendijk JA. Management of Salivary Gland Tumors. Expert Rev Anticancer Ther (2012) 12:1161–8. 10.1586/era.12.92 23098116

[36] Rizk S Robert A Vandenhooft A Airoldi M Kornek G Machiels JP. Activity of Chemotherapy in the Palliative Treatment of Salivary Gland Tumors: Review of the Literature. Eur Arch Otorhinolaryngol (2007) 264:587–94. 10.1007/s00405-007-0297-x 17415578

[37] Gu Y Lai S Wang Y Yang J Zhou P Chen T. A Population Study Comparing Tracheal and Lung Adenoid Cystic Carcinoma. Cancer Med (2024) 13:e7158. 10.1002/cam4.7158 38572933 PMC10993707

[38] Hararah MK Stokes WA Oweida A Patil T Amini A Goddard J Epidemiology and Treatment Trends for Primary Tracheal Squamous Cell Carcinoma. The Laryngoscope (2020) 130:405–12. 10.1002/lary.27994 30977524 PMC6790149

[39] Haresh K Joshi N Das P Kumar R Prabhakar R Sharma DN Unresectable Basaloid Squamous Cell Carcinoma of the Trachea Treated with Concurrent Chemoradiotherapy: A Case Report With Review of Literature. J Cancer Res Ther (2010) 6:321–3. 10.4103/0973-1482.73341 21119264

[40] Papadopoulou A Froudarakis M Abatzoglou I Koukourakis MI. Tracheal Cancer Treated With a Short Course of External and Endoluminal Radio-Chemotherapy Combined with Cetuximab - a Case Report. J Contemp Brachytherapy (2010) 2:160–2. 10.5114/jcb.2010.19496 27853478 PMC5104820

[41] Kovacs AC Vodanovich D Mogridge EK Wun L Corry J. A Case of Primary Tracheal Squamous Cell Carcinoma Arising from Malignant Transformation of Recurrent Respiratory Papillomatosis, With a Complete Response to Concurrent Chemoradiotherapy. SAGE Open Med Case Rep (2021) 9:2050313X2110546. 10.1177/2050313x211054623 PMC8543711 34707869

[42] Jiang BY Zhang JT Yan LX Chu X Nie Q Cui J Neoadjuvant Immune Checkpoint Inhibitor Plus Chemotherapy in Rare Tracheal Tumors. Cancer Commun (2021) 41:1243–5. 10.1002/cac2.12202 PMC8626609 34324788

[43] ClinicalTrials.gov. Toripalimab Combined With Chemotherapy in Primary Tracheal Squamous Cell Carcinoma; Nivolumab Combined With Chemotherapy in the Treatment of Primary Tracheal Squamous Cell Carcinoma (GALAXY-1); Nivolumab and Ipilimumab in Treating Patients With Rare Tumors. Available from: https://clinicaltrials.gov/ (Accessed May 28, 2024).

[44] Pfeffer MR Talmi Y Catane R Symon Z Yosepovitch A Levitt M. A Phase II Study of Imatinib for Advanced Adenoid Cystic Carcinoma of Head and Neck Salivary Glands. Oral Oncol (2007) 43:33–6. 10.1016/j.oraloncology.2005.12.026 16757202

[45] Chau NG Hotte SJ Chen EX Chin S Turner S Wang L A Phase II Study of Sunitinib in Recurrent And/or Metastatic Adenoid Cystic Carcinoma (ACC) of the Salivary Glands: Current Progress and Challenges in Evaluating Molecularly Targeted Agents in ACC. Ann Oncol (2012) 23:1562–70. 10.1093/annonc/mdr522 22080184

[46] Jakob JA Kies MS Glisson BS Kupferman ME Liu DD Lee JJ Phase II Study of Gefitinib in Patients With Advanced Salivary Gland Cancers. Head Neck (2015) 37:644–9. 10.1002/hed.23647 24585506 PMC5669372

[47] Locati LD Bossi P Perrone F Potepan P Crippa F Mariani L Cetuximab in Recurrent And/or Metastatic Salivary Gland Carcinomas: A Phase II Study. Oral Oncol (2009) 45:574–8. 10.1016/j.oraloncology.2008.07.010 18804410

[48] Locati LD Galbiati D Calareso G Alfieri S Singer S Cavalieri S Patients With Adenoid Cystic Carcinomas of the Salivary Glands Treated with Lenvatinib: Activity and Quality of Life. Cancer (2020) 126:1888–94. 10.1002/cncr.32754 32031693

[49] Tchekmedyian V Sherman EJ Dunn L Tran C Baxi S Katabi N Phase II Study of Lenvatinib in Patients With Progressive, Recurrent or Metastatic Adenoid Cystic Carcinoma. J Clin Oncol (2019) 37:1529–37. 10.1200/jco.18.01859 30939095 PMC6599407

[50] Kim DW Oh DY Shin SH Kang JH Cho BC Chung JS A Multicenter Phase II Study of Everolimus in Patients With Progressive Unresectable Adenoid Cystic Carcinoma. BMC Cancer (2014) 14:795. 10.1186/1471-2407-14-795 25362970 PMC4228069

[51] Ferrarotto R Mitani Y Diao L Guijarro I Wang J Zweidler-McKay P Activating NOTCH1 Mutations Define a Distinct Subgroup of Patients With Adenoid Cystic Carcinoma Who Have Poor Prognosis, Propensity to Bone and Liver Metastasis, and Potential Responsiveness to Notch1 Inhibitors. J Clin Oncol (2017) 35:352–60. 10.1200/jco.2016.67.5264 27870570 PMC5456373

[52] Even C Lassen U Merchan J Le Tourneau C Soria JC Ferte C Safety and Clinical Activity of the Notch Inhibitor, Crenigacestat (LY3039478), in an Open-Label Phase I Trial Expansion Cohort of Advanced or Metastatic Adenoid Cystic Carcinoma. Invest New Drugs (2020) 38:402–9. 10.1007/s10637-019-00739-x 30953269 PMC7066312

[53] Chahal M Pleasance E Grewal J Zhao E Ng T Chapman E Personalized Oncogenomic Analysis of Metastatic Adenoid Cystic Carcinoma: Using Whole-Genome Sequencing to Inform Clinical Decision-Making. Cold Spring Harb Mol Case Stud (2018) 4:a002626. 10.1101/mcs.a002626 29610392 PMC5880267

[54] Hotte SJ Winquist EW Lamont E MacKenzie M Vokes E Chen EX Imatinib Mesylate in Patients With Adenoid Cystic Cancers of the Salivary Glands Expressing C-Kit: A Princess Margaret Hospital Phase II Consortium Study. J Clin Oncol (2005) 23:585–90. 10.1200/jco.2005.06.125 15659505

[55] Argiris A Ghebremichael M Burtness B Axelrod RS Deconti RC Forastiere AA. A Phase 2 Trial of Bortezomib Followed by the Addition of Doxorubicin at Progression in Patients With Recurrent or Metastatic Adenoid Cystic Carcinoma of the Head and Neck: A Trial of the Eastern Cooperative Oncology Group (E1303). Cancer (2011) 117:3374–82. 10.1002/cncr.25852 21246525 PMC3135694

[56] Ho AL Dunn L Sherman EJ Fury M Baxi S Chandramohan R A Phase II Study of Axitinib (AG-013736) in Patients With Incurable Adenoid Cystic Carcinoma. Ann Oncol (2016) 27:1902–8. 10.1093/annonc/mdw287 27566443 PMC5035791

[57] Wong SJ Karrison T Hayes DN Kies M Cullen K Tanvetyanon T Phase II Trial of Dasatinib for Recurrent or Metastatic C-KIT Expressing Adenoid Cystic Carcinoma and for Nonadenoid Cystic Malignant Salivary Tumors. Ann Oncol (2016) 27:318–23. 10.1093/annonc/mdv537 26598548 PMC4722891

[58] Kim Y Lee SJ Lee JY Lee S Sun J Park K Clinical Trial of Nintedanib in Patients with Recurrent or Metastatic Salivary Gland Cancer of the Head and Neck: A Multicenter Phase 2 Study (Korean Cancer Study Group HN14-01). Cancer (2017) 123:1958–64. 10.1002/cncr.30537 28102887

[59] Goncalves PH Heilbrun LK Barrett MT Kummar S Hansen AR Siu LL A Phase 2 Study of Vorinostat in Locally Advanced, Recurrent, or Metastatic Adenoid Cystic Carcinoma. Oncotarget (2017) 8:32918–29. 10.18632/oncotarget.16464 28415633 PMC5464838

[60] Dillon PM Petroni GR Horton BJ Moskaluk CA Fracasso PM Douvas MG A Phase II Study of Dovitinib in Patients with Recurrent or Metastatic Adenoid Cystic Carcinoma. Clin Cancer Res (2017) 23:4138–45. 10.1158/1078-0432.ccr-16-2942 28377480 PMC5540767

[61] Marchioni A Tonelli R Samarelli AV Cappiello GF Andreani A Tabbì L Molecular Biology and Therapeutic Targets of Primitive Tracheal Tumors: Focus on Tumors Derived by Salivary Glands and Squamous Cell Carcinoma. Int J Mol Sci (2023) 24:11370. 10.3390/ijms241411370 37511133 PMC10379311

[62] Mosconi C de Arruda JAA de Farias ACR Oliveira GAQ de Paula HM Fonseca FP Immune Microenvironment and Evasion Mechanisms in Adenoid Cystic Carcinomas of Salivary Glands. Oral Oncol (2019) 88:95–101. 10.1016/j.oraloncology.2018.11.028 30616805

[63] Sridharan V Gjini E Liao X Chau NG Haddad RI Severgnini M Immune Profiling of Adenoid Cystic Carcinoma: PD-L2 Expression and Associations with Tumor-Infiltrating Lymphocytes. Cancer Immunol Res (2016) 4:679–87. 10.1158/2326-6066.cir-16-0031 27312343

[64] Tapias LF Shih A Mino-Kenudson M Muniappan A Gaissert HA Lanuti M Programmed Death Ligand 1 and CD8+ Immune Cell Infiltrates in Resected Primary Tracheal Malignant Neoplasms. Eur J Cardio-Thoracic Surg (2019) 55:691–8. 10.1093/ejcts/ezy370 30418532

[65] Ross JS Gay LM Wang K Vergilio J Suh J Ramkissoon S Comprehensive Genomic Profiles of Metastatic and Relapsed Salivary Gland Carcinomas Are Associated with Tumor Type and Reveal New Routes to Targeted Therapies. Ann Oncol (2017) 28:2539–46. 10.1093/annonc/mdx399 28961851 PMC5834110

[66] Cohen RB Delord JP Doi T Piha-Paul SA Liu SV Gilbert J Pembrolizumab for the Treatment of Advanced Salivary Gland Carcinoma: Findings of the Phase 1b KEYNOTE-028 Study. Am J Clin Oncol (2018) 41:1083–8. 10.1097/coc.0000000000000429 29462123 PMC6211783

[67] Mahmood U Bang A Chen YH Mak RH Lorch JH Hanna GJ A Randomized Phase 2 Study of Pembrolizumab With or without Radiation in Patients with Recurrent or Metastatic Adenoid Cystic Carcinoma. Int J Radiat Oncology*Biology*Physics (2021) 109:134–44. 10.1016/j.ijrobp.2020.08.018 PMC9361179 32781104

[68] Network NCC. Head and Neck Cancers (Version 3.2024) (2024). Available from: https://www.nccn.org/professionals/physician_gls/pdf/head-and-neck.pdf (Accessed May 5, 2024).

[69] Maio M Ascierto PA Manzyuk L Motola-Kuba D Penel N Cassier P Pembrolizumab in Microsatellite Instability High or Mismatch Repair Deficient Cancers: Updated Analysis from the Phase II KEYNOTE-158 Study. Ann Oncol (2022) 33:929–38. 10.1016/j.annonc.2022.05.519 35680043

[70] Mikami E Nakamichi S Nagano A Misawa K Hayashi A Tozuka T Successful Treatment with Definitive Concurrent Chemoradiotherapy Followed by Durvalumab Maintenance Therapy in a Patient with Tracheal Adenoid Cystic Carcinoma. Intern Med (2023) 62:2731–5. 10.2169/internalmedicine.1142-22 36642523 PMC10569923

[71] Maller B Kaszuba F Tanvetyanon T. Complete Tumor Response of Tracheal Squamous Cell Carcinoma After Treatment With Pembrolizumab. The Ann Thorac Surg (2019) 107:e273–e274. 10.1016/j.athoracsur.2018.08.067 30326234

[72] Osho AA Azzoli CJ Pai S Mino-Kenudson M Faquin WC Huynh TG Successful Treatment of an Aggressive Tracheal Malignancy with Immunotherapy. The Ann Thorac Surg (2017) 103:e123–e125. 10.1016/j.athoracsur.2016.08.021 28109369 PMC7388728

[73] Honings J Gaissert HA van der Heijden HF Verhagen AF Kaanders JH Marres HA. Clinical Aspects and Treatment of Primary Tracheal Malignancies. Acta Oto-Laryngologica (2010) 130:763–72. 10.3109/00016480903403005 20569185

[74] Nilssen Y Solberg S Brustugun OT Møller B Sundset A Wahl SGF Tracheal Cancer: A Rare and Deadly but Potentially Curable Disease that Also Affects Younger People. Eur J cardio-thoracic Surg official J Eur Assoc Cardio-thoracic Surg (2023) 64:ezad244. 10.1093/ejcts/ezad244 PMC10329490 37348858

[75] Szyfter W Wierzbicka M Popko M Pastusiak T Balcerowiak A. Management of Laryngo-Tracheal Stenosis. Kardiochir Torakochirurgia Pol (2009) 6:157–65.

[76] Joshi N Mallick S Haresh KP Gandhi A Prabhakar R Laviraj M Modern Chemoradiation Practices for Malignant Tumors of the Trachea: An Institutional Experience. Indian J Cancer (2014) 51:241–4. 10.4103/0019-509x.146743 25494113

[77] Videtic GM Campbell C Vincent MD. Primary Chemoradiation as Definitive Treatment for Unresectable Cancer of the Trachea. Can Respir J (2003) 10:143–4. 10.1155/2003/382026 12712222

[78] Allen AM Rabin MS Reilly JJ Mentzer SJ. Unresectable Adenoid Cystic Carcinoma of the Trachea Treated With Chemoradiation. J Clin Oncol (2007) 25:5521–3. 10.1200/jco.2007.13.7273 18048830

[79] Yathiraj PH Ail S Singh A Mamidipudi V. Unresectable Squamous Cell Carcinoma of Upper Trachea With Long-Term Survival after Concurrent Chemoradiotherapy. BMJ Case Rep (2017) 2017:bcr2017221284–2017-221284. 10.1136/bcr-2017-221284 PMC5747826 28801512

[80] Larnaudie A Orliac H Lerouge D Naessens C Deny N Serre R Radiotherapy for Tracheal Squamous Cell Carcinoma: A Review. Arch Clin Med Case Rep (2022) 6:653–59. 10.26502/acmcr.96550539

[81] Yan M Louie AV Kotecha R Ashfaq Ahmed M Zhang Z Guckenberger M Stereotactic Body Radiotherapy for Ultra-Central Lung Tumors: A Systematic Review and Meta-Analysis and International Stereotactic Radiosurgery Society Practice Guidelines. Lung Cancer (2023) 182:107281. 10.1016/j.lungcan.2023.107281 37393758

[82] Laramore GE Krall JM Griffin TW Duncan W Richter MP Saroja KR Neutron versus Photon Irradiation for Unresectable Salivary Gland Tumors: Final Report of an RTOG-MRC Randomized Clinical Trial. Int J Radiat Oncology*Biology*Physics (1993) 27:235–40. 10.1016/0360-3016(93)90233-l 8407397

[83] Zeng R Wang H Cai X Guo X Ping Y Yang Q. Radiotherapy for Primary Tracheal Carcinoma: Experience at a Single Institution. Technol Cancer Res Treat (2021) 20:153303382110342. 10.1177/15330338211034273 PMC8361538 34372715

[84] Hogerle BA Lasitschka F Muley T Bougatf N Herfarth K Adeberg S Primary Adenoid Cystic Carcinoma of the Trachea: Clinical Outcome of 38 Patients After Interdisciplinary Treatment in a Single Institution. Radiat Oncol (2019) 14:117. 10.1186/s13014-019-1323-z 31272473 PMC6610895

[85] Tun AJ Hoppe BS Zhao Y Makey I Fernandez-Bussy S Liang X. Radiation Therapy for Primary Adenoid Cystic Carcinoma of the Trachea: Photons, Protons, or Carbon. Int J Part Ther (2023) 9:302–5. 10.14338/ijpt-22-00036.1 37169012 PMC10166014

