# Supplementary material for: Tracheal Tumors: Clinical Practice Guidelines for Palliative Treatment and Follow-Up
Source: Oncol Rev. 2024 Sep 18;18:1451247. doi: 10.3389/or.2024.1451247 (PMC11445028; doi:10.3389/or.2024.1451247)
Supplement: Supplementary file 1 [file Table1.DOCX]

Supplementary LoE/GoR table

Table 1. Levels of evidence and grades of recommendation (adapted from the Infectious Diseases Society of America-United States Public Health Service Grading Systema)

Levels of evidence

| I | Evidence from at least one large randomised, controlled trial of good methodological quality (low potential for bias) or meta-analyses of well- conducted randomised trials without heterogeneity |
| --- | --- |
| II | Small randomised trials or large randomised trials with a suspicion of bias (lower methodological quality) or meta-analyses of such trials or of trials with demonstrated heterogeneity |
| III | Prospective cohort studies |
| IV | Retrospective cohort studies or case-control studies |
| V | Studies without control group, case reports, expert opinions |

Grades of recommendation

| A | Strong evidence for efficacy with a substantial clinical benefit, strongly recommended |
| --- | --- |
| B | Strong or moderate evidence for efficacy but with a limited clinical benefit, generally recommended |
| C | Insufficient evidence for efficacy or benefit does not outweigh the risk or the disadvantages (adverse events, costs, etc.), optional |
| D | Moderate evidence against efficacy or for adverse outcome, generally not recommended |
| E | Strong evidence against efficacy or for adverse outcome, never recommended |
